# Impaired phosphate transport in *SLC34A2* variants in patients with pulmonary alveolar microlithiasis

**DOI:** 10.1186/s40246-022-00387-y

**Published:** 2022-04-20

**Authors:** Åsa Lina M. Jönsson, Nati Hernando, Thomas Knöpfel, Susie Mogensen, Elisabeth Bendstrup, Ole Hilberg, Jane Hvarregaard Christensen, Ulf Simonsen, Carsten A. Wagner

**Affiliations:** 1 Department of Biomedicine, Aarhus University, Aarhus, Denmark; 2 Department of Clinical Genetics, Aarhus University Hospital, Aarhus, Denmark; 3 Institute of Physiology, University of Zurich, Zurich, Switzerland; 4 Swiss National Center of Competence in Research NCCR Kidney.CH, Zurich, Switzerland; 5 Centre for Rare Lung Diseases, Department of Respiratory Diseases and Allergy, Aarhus University Hospital, Aarhus, Denmark; 6 Medical Department, Vejle Hospital, Vejle, Denmark

**Keywords:** Pulmonary alveolar microlithiasis (PAM), Sodium-dependent phosphate transport protein 2B, NaPi-IIb, Sodium-dependent phosphate transport protein 2B, NaPi-2b, *SLC34A2*, *SLC34A2* variants, *SLC34A2* mutations, In-frame deletion, frameshift variant, nonsense variant, *Xenopus laevis* oocytes

## Abstract

**Background:**

Variants in *SLC34A2* encoding the sodium-dependent phosphate transport protein 2b (NaPi-IIb) cause the rare lung disease pulmonary alveolar microlithiasis (PAM). PAM is characterised by the deposition of calcium-phosphate concretions in the alveoli usually progressing over time. No effective treatment is available. So far, 30 allelic variants in patients have been reported but only a few have been functionally characterised. This study aimed to determine the impact of selected *SLC34A2* variants on transporter expression and phosphate uptake in cellular studies.

**Methods:**

Two nonsense variants (c.910A > T and c.1456C > T), one frameshift (c.1328delT), and one in-frame deletion (c.1402_1404delACC) previously reported in patients with PAM were selected for investigation. Wild-type and mutant c-Myc-tagged human NaPi-IIb constructs were expressed in *Xenopus laevis* oocytes. The transport function was investigated with a ^32^Pi uptake assay. NaPi-IIb protein expression and localisation were determined with immunoblotting and immunohistochemistry, respectively.

**Results:**

Oocytes injected with the wild-type human NaPi-IIb construct had significant ^32^Pi transport compared to water-injected oocytes. In addition, the protein had a molecular weight as expected for the glycosylated form, and it was readily detectable in the oocyte membrane. Although the protein from the Thr468del construct was synthesised and expressed in the oocyte membrane, phosphate transport was similar to non-injected control oocytes. All other mutants were non-functional and not expressed in the membrane, consistent with the expected impact of the truncations caused by premature stop codons.

**Conclusions:**

Of four analysed *SLC34A2* variants, only the Thr468del showed similar protein expression as the wild-type cotransporter in the oocyte membrane. All mutant transporters were non-functional, supporting that dysfunction of NaPi-IIb underlies the pathology of PAM.

**Supplementary Information:**

The online version contains supplementary material available at 10.1186/s40246-022-00387-y.

## Background

**Fig. 1:**
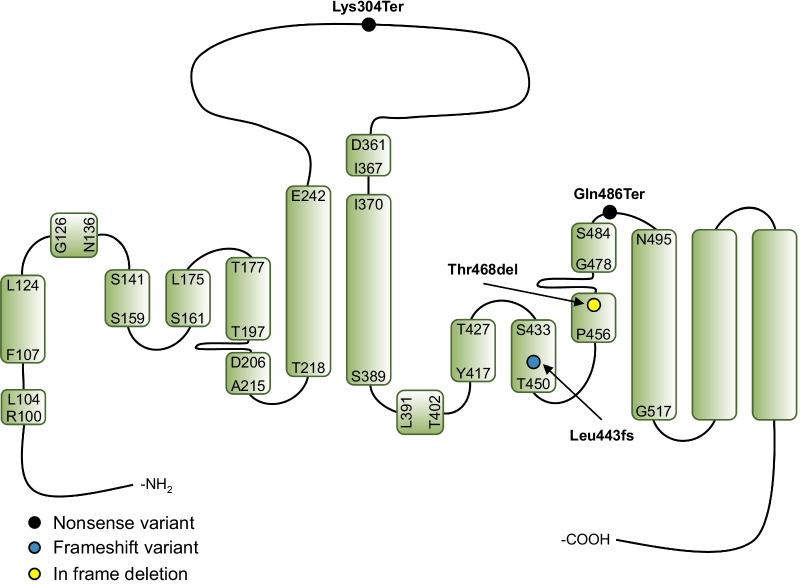
Predicted model of the secondary topology of the human NaPi-IIb protein; the locations of the four investigated variants are indicated. The current model of SLC34 transporters based on homology modeling with the bacterial dicarboxylate cotransporter VcINDY as a template, was adapted from the model of the predicted structure of human NaPi-IIa by Fenollar-Ferrer et al. [12]. The Na1-binding site is presumed to be formed by the amino acids T197 (Thr), Q203 (Gln), D206 (Asp), N224 (Asn), and S461 (Ser), the Na2-binding site by S161 (Ser), T192 (Thr), S193 (Ser), and N196 (Asn), the Pi-binding site by S161 (Ser) and S433 (Ser), and the Na3-binding site by Q431 (Gln), S432 (Ser), S433 (Ser), T465 (Thr), and T468 (Thr) [13]. The protein sequences used for alignment in Clustal Omega version 1.2.4 [14]: Ensembl Transcript ID ENST00000382051.7 and Transcript ID ENST00000324417.5 release 92 (Human (GRCh38.p12) assembly), and Ensembl Transcript ID ENSRNOT00000033749.5 (Rat (Rnor_6.0) assembly). A (Ala), R (Arg), N (Asn), D (Asp), E (Glu), G (Gly), I (Ile), L (Leu), P (Pro), F (Phe), S (Ser), T (Thr), Y (Tyr)

Pulmonary alveolar microlithiasis (PAM) (OMIM #265100) is a rare autosomal recessive disease characterised by the formation of calcium-phosphate concretions (microliths) in the alveoli of the lungs [1]. Usually, the disease progresses slowly and often leads to respiratory failure. Currently, lung transplantation is the only effective treatment [2]. PAM is caused by genetic variants in *SLC34A2* (Entrez Gene ID 10568) encoding the sodium (Na^+^)-dependent phosphate (Pi) transport protein 2B, NaPi-IIb (NP_006415). *SLC34A2* is located on the short arm of chromosome 4 (4p15.2) and consists of 12 coding exons. The 690 amino acid protein NaPi-IIb is one of the members of the solute carrier family SLC34, which furthermore consists of NaPi-IIa (*SLC34A1*) and NaPi-IIc (*SLC34A3*) [3–6]. These cotransporters are responsible for maintaining whole-body phosphate homeostasis by regulating renal (NaPi-IIa, NaPi-IIc) and intestinal (NaPi-IIb) phosphate transport [7]. All three isoforms use the Na^+^ electrochemical gradient to mediate divalent phosphate transport (HPO_4_^2−^) against its concentration gradient. NaPi-IIa and NaPi-IIb are both electrogenic transporters. They are characterised by a transport stoichiometry of 3:1 (Na^+^:HPO_4_^2−^), with one net positive charge per transport cycle [8]. The SLC34 transporters share high sequence similarity, and all eukaryotic NaPi-II homologs are assumed to have similar transmembrane (TM) topology and expected to share a common 3-D structure [9, 10]. The NaPi-II proteins contain two inverted repeated units of conserved sequence connected by a large extracellular loop, and with both termini located in the cytosol (Fig. 1) [7, 11].

NaPi-IIb mRNA is expressed in several organs, including the small intestine, mammary gland, kidney, and liver, and highly expressed in type II alveolar cells in the lungs [15–19]. NaPi-IIb may play an important role in the regulation of Pi levels in the fluid lining the alveolar space [18, 20]. Dysfunction of NaPi-IIb due to variants in *SLC34A2* may thus lead to decreased cell-uptake of Pi and increased levels in the alveolar space followed by precipitation with extracellular calcium, i.e., crystal formation [21].

Since identifying *SLC34A2* as a disease causative gene in 2006 [21, 22], 30 allelic variants have been reported in PAM patients [1]. Most changes involve a single or a few nucleotides in coding exons, but larger deletions, an indel variant, and changes at the promoter and splice sites have also been reported [1]. Functional studies investigating the impact of *SLC34A2* variants are very sparse. Two variants, predicted to result in a truncated protein, were found non-functional in Pi uptake when expressed in *Xenopus* oocytes [21]. In addition, a missense variant expressed in a human alveolar epithelial cell line (A549 cells) revealed evidence of dysfunctional phosphate transport [23].

The present study aimed to investigate the transport function and the cellular expression of a subset of genetic variants in *SLC34A2* identified in patients with PAM.

## Results

### Genetic variants of *SLC34A2* are located in conserved regions

**Fig. 2:**
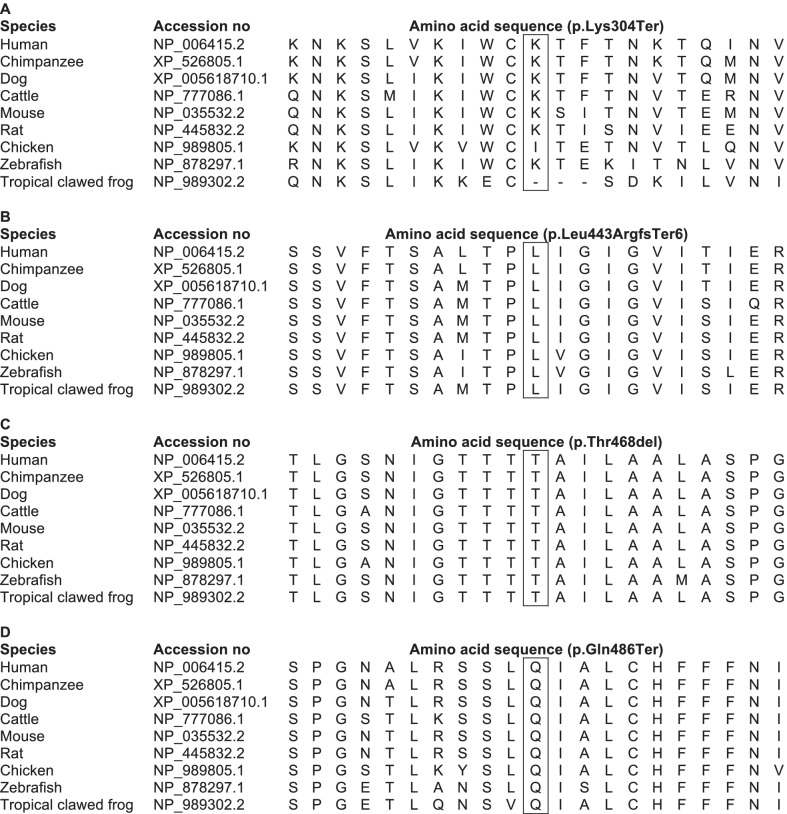
Evolutionary conservation of the K304 (Lys), L443 (Leu), T468 (Thr), and Q486 (Gln) residues. Partial amino acid sequences encoded by exon 8 (Panel **A**), exon 11 (Panel **B**), and exon 12 (Panel **C** and **D**) are shown. The residues mutated in the patients are boxed. A (Ala), R (Arg), N (Asn), D (Asp), C (Cys), Q (Gln), E (Glu), G (Gly), H (His), I (Ile), L (Leu), K (Lys), M (Met), F (Phe), P (Pro), S (Ser), T (Thr), W (Trp), Y (Tyr), V (Val)

We selected four *SLC34A2* variants previously reported in patients with PAM [22, 24–30] to investigate their effects on Pi transport function and cellular expression of NaPi-IIb. Variants located in presumably conserved regions of the protein and variants only involving a few nucleotides or amino acids were considered relevant for the investigation. Table 1 summarises the main characteristics of the *SLC34A2* variants investigated. They included an in-frame single amino acid deletion (Thr468del) in a conserved region of NaPi-IIb encoded by exon 12 along with two nonsense (Lys304Ter and Gln486Ter) and one frameshift variant (Leu443fs), the latter three all associated with introduction of premature termination codons (PTCs) in different gene contexts. These were, respectively, 18 base pairs upstream of the exon 8–9 junction (Lys304Ter), 114 base pairs upstream of the exon 11–12 junction (Leu443ArgfsTer6), and 3 base pairs upstream of the exon 12–13 (last exon) junction (Gln486Ter). The predicted location of the affected sites in the NaPi-IIb model is shown in Fig. 1. All the primary mutated amino acids (Lys304, Leu443, Thr468, and Gln486) are conserved among at least six different mammalian and non-mammalian species (Fig. 2). Thr468del is predicted to affect the transmembrane (TM) domain 5a of NaPi-IIb. In case, the three PTCs would escape nonsense mediated decay (NMD) of the *SLC34A2* mRNA, they would result in variable sized truncations of the NaPi-IIb C-terminal. Table 2 summarises the properties of the investigated NaPi-IIb constructs.

**Table 1:** Overview of the *SLC34A2* variants investigated

Construct	Nucleotide change	Protein change	Patient origin	References
Lys304Ter	c.910A > T	p.Lys304Ter	China	[24, 25, 29]
Leu443fs	c.1328delT	p.Leu443ArgfsTer6	Turkey and Morocco	[22, 30]
Thr468del	c.1402_1404delACC	p.Thr468del	Denmark and USA*	[26, 28]
Gln486Ter	c.1456C > T	p.Gln486Ter	Turkey	[27]

*Originally from Italy. *SLC34A2* DNA reference sequence: Ensembl Transcript ID ENST00000382051.7 (GRCh38.p12 assembly)

**Table 2:** Properties of human c-Myc-NaPi-IIb constructs

Predicted location in the human NaPi-IIb protein*	Construct	^32^Pi uptake	Expression	
			WB	IC
	WT	+	+	+
Long EC-Loop (truncation of amino acids 304–690)	Lys304Ter	−	+	−
HP2b (mutation of amino acids 443–448 and truncation of amino acids 449–690)	Leu443fs	−	+	−
TM domain 5a	Thr468del	−	+	+
Short EC-Loop btw. TM domains 5b and 6 (truncation of amino acids 486–690)	Gln486Ter	−	+	−

*btw* between, *EC* extracellular, *HP* Hairpin, *IC* immunocytochemistry, *TM* transmembrane, *VcINDY* Na^+^-coupled dicarboxylate transporter from Vibrio cholera, *WB* Western blot (oocyte lysate), *WT* wild-type c-Myc-hNaPi-IIb, + positive, − negative; *The protein model is based on homology modelling with the bacterial dicarboxylate cotransporter VcINDY as template, adapted from the model of the predicted structure of human NaPi-IIa by Fenollar-Ferrer et al. [12]. *SLC34A2* DNA reference sequence: Ensembl Transcript ID ENST00000382051.7 (GRCh38.p12 assembly)

### Impaired ^32^Pi transport of hNaPi-IIb mutants

**Fig. 3:**
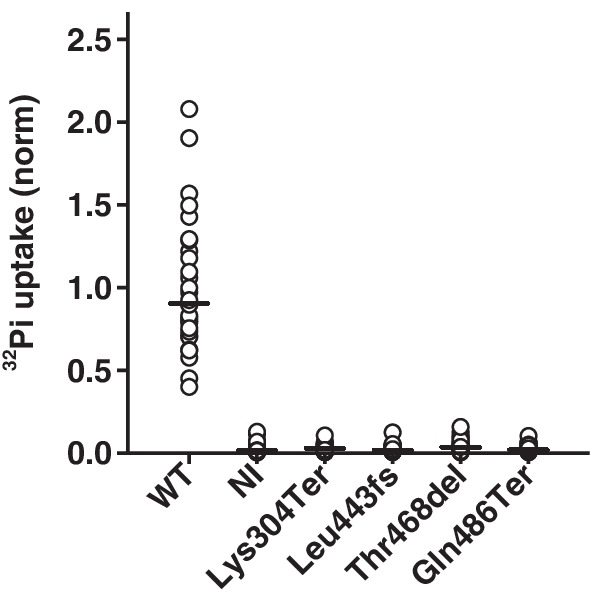
Phosphate transport activity in *Xenopus laevis* oocytes 3 days after injection of cRNA encoding wild-type (WT) or mutated c-Myc-tagged hNaPi-IIb constructs. NI is non-injected oocytes serving as additional controls. Data presented are values from 3 to 4 independent experiments with 7–10 oocytes per group incubated for 10 min in ND96 containing 1 mM cold Pi and ^32^P. Vertical bars represent the median transport activity. There was a statistically significant between-group difference (*p* = 0.0001, Kruskal–Wallis test by ranks). Post-hoc analysis revealed a statistically significant difference between WT and both mutants and NI control oocytes (*p* < 0.05, Dunn's multiple comparison test). ^32^Pi uptake levels in oocytes expressing mutants were similar to non-injected oocytes (*p* > 0.05, Dunn's multiple comparison test)

As expected, expression of wild-type (WT) human NaPi-IIb (hNaPi-IIb) in *Xenopus laevis* oocytes led to a statistically significant (*p* = 0.001) increase in ^32^Pi uptake compared to water-injected oocytes (Additional file 1: Fig. S1). NaPi-IIb mutants Lys304Ter, Leu443fs, Thr468del, and Gln486Ter, did not induce transport of Pi and showed a similar uptake level to non-injected controls (Fig. 3). These findings demonstrate that none of the mutants was able to induce measurable ^32^Pi uptake.

### Mutants affect NaPi-IIb protein expression

**Fig. 4:**
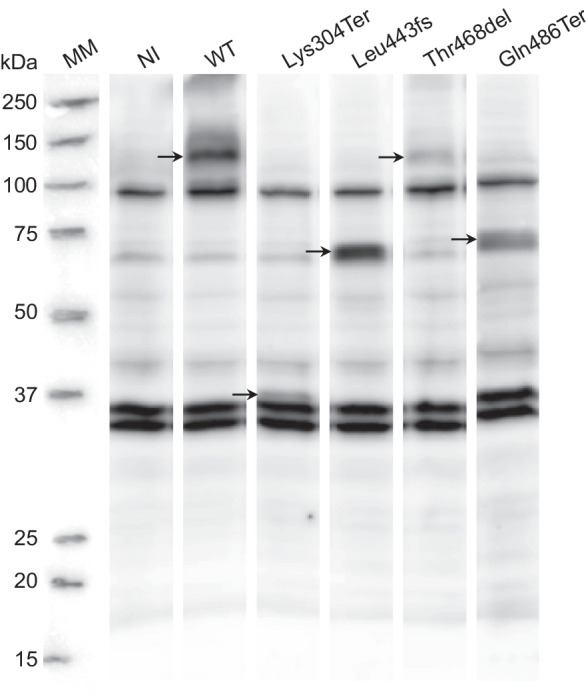
Molecular identification of c-Myc hNaPi-IIb expression by Western Blot of lysates from *Xenopus laevis* oocytes injected with wild-type (WT) and mutated constructs. Each lane was loaded with a volume corresponding to one oocyte. Lanes correspond to (NI) non-injected control oocytes, (WT) wild type, Lys304Ter, Leu443fs, Thr468del, and Gln486Ter. Blots were incubated with a monoclonal c-Myc-specific antibody. The approximate protein molecular mass is indicated in kilodaltons (kDa). Specific immunoreactive protein bands are detected at ∼ 110–125 kDa for WT and Thr468del, at ∼ 35 kDa for Lys304Ter, and at ∼ 60 kDa for Leu443fs and Gln486Ter

To assess if the reduction in Pi transport observed in the mutants was caused by reduced or absent protein expression, immunoblotting of total oocyte lysates was performed using a c-Myc antibody. NaPi-IIb is a 690 amino acid protein with an expected molecular weight of about 75 kDa. However, the cotransporter is detected as a smear above 110 kDa in Western blots of intestinal brush border membranes (BBM) isolated from adult mice and in crude membrane fractions from the lungs of mice [4, 18, 31–33]. Digestion of intestinal BBM with PNGase F shifts the apparent molecular size to the expected 75 kDa, suggesting that NaPi-IIb is a N-linked glycoprotein [32]. Figure 4 shows a Western blot of cell lysates from control oocytes and oocytes expressing WT and mutant NaPi-IIb proteins (original Western Blot is presented in Additional file 1: Fig. S2). Similar to murine intestinal and lung tissue, a ∼ 110–125 kDa band was detected in samples from oocytes injected with the WT cotransporter, consistent with the molecular weight of glycosylated NaPi-IIb protein. No signal at this position was detectable in the control lane loaded with lysates from non-injected oocytes, indicating that the signal in the wells loaded with samples from WT-injected oocytes was specific for NaPi-IIb. Only one mutant (Thr468del) showed a band of a similar size as the WT cotransporter. Lower molecular weight bands at ∼ 35–60 kDa were observed for all other mutants; these roughly corresponding to the expected size of the individual truncated protein products (Lys304Ter: 33 kDa, Leu443fs: 49 kDa, and Gln486Ter: 53 kDa). The c-Myc tag consists of 10 amino acids (∼ 1.1 kDa), which corresponds to approximately 0.9–1.0% and 1.4% of the predicted molecular weight of glycosylated and unglycosylated NaPi-IIb protein, respectively. These findings indicate that only the three-nucleotide deletion Thr468del was expressed with the correct size at the protein level.

### NaPi-IIb variants alter subcellular localisation

**Fig. 5:**
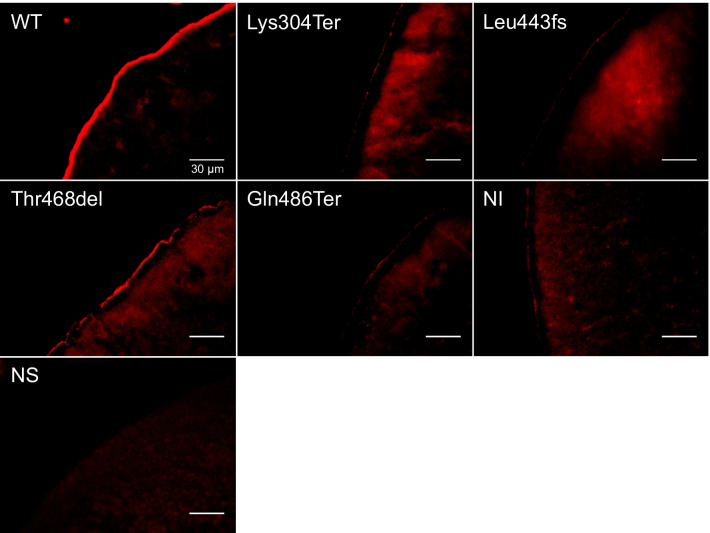
Immunofluorescence in *Xenopus laevis* oocytes expressing wild-type c-Myc-hNaPi-IIb (WT), mutants (Lys304Ter, Leu443fs, Thr468del, and Gln486Ter), non-injected (NI) control oocytes, and oocytes with no secondary antibody (NS). Immunostaining confirms cell membrane localisation of WT and Thr468del c-Myc-hNaPi-IIb constructs. Cryosections of oocytes were incubated with monoclonal c-Myc antibody. Scale bar: 30 µm. Magnification: × 63

A fluorescence signal at the cell membrane was observed in oocytes injected with WT hNaPi-IIb cRNA (Fig. 5), indicating membrane expression of the WT Myc-fused cotransporter. A similar pattern was found in cells injected with the T468del construct. However, no significant signal was detectable at the membrane in oocytes expressing the Lys304Ter, Leu443fs, and Gln486Ter constructs and non-injected controls. These findings suggest that the three-nucleotide deletion Thr468del was the only mutant able to stably reach the membrane.

## Discussion

To determine the impact of *SLC34A2* variants on the transport function of NaPi-IIb, c-Myc-tagged transporters were expressed in *Xenopus laevis* oocytes and investigated for ^32^Pi uptake, protein expression, and membrane location. Four variants previously found in patients with PAM were constructed and investigated together with WT hNaPi-IIb. The selected variants were expected to have different effects on the protein and affect the cotransporter at different locations. Both truncating variants and a small in-frame variant were represented. Investigation of several different variant types made it possible to compare their impact on the transport function. The WT cotransporter mediated significant phosphate uptake, whereas the uptake from all mutants was indistinguishable from non-injected control oocytes. Protein expression was confirmed in all mutants but with an apparent correct synthesis of the fully glycosylated protein, a WT-like expression, only in one construct (Thr468del). The same mutant was also expressed in the oocyte membrane. This indicates that the Thr468del mutant does not function properly or may not be sufficiently expressed in the membrane, or a combination of both.

No genotype–phenotype correlation exists in PAM, although a correlation was found between disease severity and genotype in a previous report of 14 patients [28]. Adult patients, homozygous for the variants investigated in this study, were all previously reported to be symptomatic [22, 25, 26, 28–30]. The c.1456C > T has only been reported in an asymptomatic 12-year-old girl [27]. Both c.910A > T and c.1402_1404del are reported in at least three unrelated patients and may therefore be relatively common in PAM [24–26, 28, 29]. The c.910A > T is further reported in a symptomatic adult patient in a compound heterozygous state with a missense variant on the other allele [34].

The pattern of migration of WT hNaPi-IIb and mutant constructs expressed in oocytes was assessed by Western blot of oocyte lysates. Since the c-Myc tag only consists of 10 amino acids (∼ 1.1 kDa), it was not expected to impact the predicted molecular weight of hNaPi-IIb. The anti-c-Myc antibody detected a main specific band of about 110–125 kDa in oocytes injected with the tagged WT construct. This is in accordance with the expected size of the fully functional and glycosylated NaPi-IIb protein as previously reported in the lung tissue [18, 35] and small intestine [31, 35, 36] in mice. Only one mutant construct (Thr468del) migrated as a band with the similar size. This mutant contained a three-nucleotide deletion, resulting in deletion of Thr468 but without altering the open reading frame. As expected, it did not significantly change the protein expression pattern in Western Blot. As expected, the other three mutants were not expressed in full length since the variants all carried PTCs at different positions.

Immunofluorescence confirmed oocyte membrane expression of the Thr468del mutant, although it seemed to be reduced compared to the WT. Amino acids 465–468 are presumably located close to the predicted substrate-binding domain site (Na3- or Pi-binding site) [11, 13]. Deleting an amino acid in this area might cause substantial structural changes in the protein configuration leading to abnormal folding and impaired membrane integration or even direct degradation. This conformational change might completely disrupt the transport pathway or possibly lead to altered kinetics of the transporter.

The deleted Thr468 is located in an area of four subsequent threonine residues. In a previous study of rat NaPi-IIa using the substituted cysteine accessibility method (SCAM), substitution included the amino acid position corresponding to the Thr468 in NaPi-IIb [37]. Interestingly, when the first threonine residue was substituted with cysteine, the fully glycosylated cotransporter protein was not synthesised. The protein was functionally inactive with no electrogenic activity and ^32^Pi uptake at the same level as the background. In contrast, a fully synthetised and functional protein was revealed when the third threonine residue was substituted. Another study of human NaPi-IIa included the analysis of the first and last threonine residue [13]. When the amino acids were replaced by alanine, both mutant constructs were expressed in the oocyte membrane. However, the constructs were non-functional with no Pi-induced inward currents, and ^32^Pi uptake was indistinguishable from the background level. Thus, the region harbouring Thr468 is critical to maintain normal transporter function. One previous study reported functional data of two truncated mutants found in patients with PAM and expressed in *Xenopus* oocytes [21]. The variants included a complex deletion-insertion (c.857_871delins19), causing a frameshift with a PTC, and a splice-site variant at the 5′ end of intron 9 (c.1048 + 1G > A). When expressed in oocytes, both variants were unable to transport phosphate and no inward current was registered.

Our study has some limitations including the analysis of only a subset of known PAM-associated variants, lack of investigations on the gene context dependent effects of the variants on NMD, the focus on *Xenopus* oocytes as heterologous expression system, and absence of data on the intracellular fate of most variants that could not be detected by immunofluorescence or functional assays. The use of mammalian cell models to express normal and mutant NaPi-IIb proteins and investigation of the variants inducing PTCs in their natural gene context may help answer some of these questions. Overall, further characterisation of variants in *SLC34A2* identified in patients with PAM is needed to improve understanding of the PAM pathophysiology and test for genotype–phenotype associations. Such investigations may also contribute to develop interventions in a disease with no known effective therapy except lung transplantation.

## Conclusions

In conclusion, our results demonstrate that expression of truncated NaPi-IIb caused by the Lys304Ter, Leu443fs, and Gln486Ter variants affect critical structures in the NaPi-IIb transporter. These alterations revealed protein products with defective trafficking unable to get targeted to the membrane, thus leading to loss of transport function. The Thr468del mutant displayed no measurable functionality even though it was correctly synthesised and inserted into the oocyte membrane. Our findings support the underlying dysfunction of NaPi-IIb in PAM.

## Methods

### Variants and preparation of plasmid constructs

The variants investigated were two nonsense, one frameshift, and one in-frame deletion: c.910A > T (p.Lys304Ter) [24, 25, 29], c.1328delT (p.Leu443ArgfsTer6) [22, 30], c.1402_1404delACC (p.Thr468del) [26, 28], and c.1456C > T (p.Gln486Ter) [27]. These changes were introduced into human *SLC34A2* cDNA and subcloned into a KSM vector. All constructs were fused N-terminally to c-Myc.

Mutagenic primers were designed using the Jellyfish Version 1.0 software programme. The variants were introduced into a commercially available c-Myc-DDK-tagged *SLC34A2* vector, in which the c-Myc epitope was fused to the C-terminus of the cotransporter in a pCMV6 backbone (OriGene Technologies, Rockville, MD, USA) (NCBI GenBank accession no. NM_006424 and AF067196), using the QuickChange site-directed mutagenesis kit (Stratagene, La Jolla, CA, USA). The plasmids containing generated variants were isolated using QIAprep Spin Miniprep Kit (QIAGEN, 27106), and all variants were verified by sequencing (Eurofins Genomics, Ebersberg, Germany).

Because the C-terminus of other SLC34 transporters is required for proper sorting, plasmids containing the c-Myc epitope fused to the N-terminal tail of the transporter were then generated using the above constructs as templates. Thus, complementary oligonucleotides containing the Kozak consensus sequence followed by the sequence encoding the c-Myc (Microsynth AG, Balgach, Switzerland) were first annealed and purified with the QIAEX II Gel Extraction Kit (QIAGEN). The annealed fragment contained HindIII (upstream) and EcoRI (downstream) restriction sites. Upon annealing and digestion with HindIII and EcoRI (Thermo Scientific) the c-Myc sequence was ligated with T4 DNA Ligase (Thermo Scientific) into a KSM expression vector (previously digested with the same enzymes) that contains the 5′ and 3′ UTRs from Xenopus β-globin to optimise its expression in oocytes as described by Virkki et al. [38]. Hereafter, the ligation product was transfected into competent cells. Plasmid DNA was purified with QIAprep Spin Miniprep Kit (QIAGEN, 27106) and sequenced (Microsynth AG, Balgach, Switzerland) to confirm the presence of the c-Myc sequence.

The cDNAs encoding hNaPi-IIb (WT and mutants) were amplified from the original vector (pCMV6 backbone) by PCR. The sense primer contained an EcoRI site, whereas the antisense primer included a stop codon downstream of the last amino acid of the cotransporter followed by a BamHI site. After purification by use of the QIAquickPCR purification kit (QIAGEN), NaPi-IIb WT and mutants cDNAs were cut from the vector by EcoRI and BamHI digestion (Thermo Scientific). The isolated hNaPi-IIb inserts were then ligated into the c-Myc-tagged KSM vector (Additional file 1: Fig. S3) previously digested with the same enzymes and transfected into competent cells. After purification, KSM plasmids containing c-Myc-tagged WT and mutated hNaPi-IIb cDNA inserts were verified by DNA sequencing (Microsynth, AG, Balgach, Switzerland). For oocyte expression, plasmids were linearised with NotI (Thermo Scientific), and cRNA was synthesised in the presence of a cap analogue using the Megascript T3 kit (Ambion). The RNA was purified using the RNeasy Mini Kit (QIAGEN). After measuring the concentration by spectrophotometry, the samples were diluted to 0.2 µg/µl in nuclease-free water.

### Expression of hNaPi-IIb wild type and mutants in *Xenopus laevis* oocytes

Oocytes were harvested from *Xenopus laevis* frogs anesthetised in MS222 (tricaine methansulphonate). Oocytes were treated with 1 mg/ml collagenase (Type I-A, Sigma-Aldrich, Buchs, Switzerland) in 100Na solution (100 mM NaCl, 2 mM KCl, 1 mM MgCl_2_, and 10 mM HEPES, pH 7.4 adjusted with Tris) in the presence of 0.1 mg/ml trypsin inhibitor (Type III-O, Sigma-Aldrich, Buchs, Switzerland). The oocytes were stored in modified Barth’s solution (88 mM NaCl, 1 mM KCl, 0.41 mM CaCl_2_, 0.82 mM MgSO_4_, 2.5 mM NaHCO_3_, 2 mM Ca(NO_3_)_2_, 7.5 mM HEPES, pH 7.5 adjusted with Tris) supplemented with antibiotics (doxycycline and gentamicin, 5 mg/l each). Healthy stage V–VI oocytes were selected and incubated in ND96 solution (96 mM NaCl, 2 mM KCl, 1.8 mM CaCl_2_, 1 mM MgCl_2_, 5 mM Hepes, pH 7.4 adjusted with Tris) supplemented with antibiotics (doxycycline and gentamicin, 5 mg/l each). The oocytes were injected by use of the Nanoject II (Drummond Scientific Company, Broomall, USA), with 50 nl of in vitro synthesised cRNA (0.2 mg/ml) and maintained at 16 °C in ND96 solution supplemented with doxycycline and gentamicin (5 mg/l each). [^32^P] phosphate flux measurements were performed 3 days after injection. Non-injected and water-injected oocytes served as controls. All animal procedures were conducted following the Swiss Cantonal and Federal legislation for experiments involving animals. All standard reagents were obtained from Fluka (Buchs, Switzerland).

### ^32^Pi uptake studies in ***Xenopus laevis*** oocytes

Non-injected control oocytes, oocytes expressing WT hNaPi-IIb, and mutants (7–10 oocytes per group) were first equilibrated in ice-cold ND96 without a tracer. After aspiration of the solution, the oocytes were incubated in ND96 containing 1 mM cold Pi and ^32^P (specific activity 10 mCi/mmol Pi) for 10 min (see below) at 25 °C. After incubation, the solution was removed, and oocytes were washed four times with ice-cold ND96. Each oocyte was transferred to a scintillation vial and lysed in 200 μl of 2% sodium dodecyl sulfate (SDS) for 45 min by shaking before the addition of 3 ml scintillation fluid (Emulsifier-Safe, PerkinElmer). The amount of radioactivity accumulated in each oocyte was measured by scintillation counting in a β-counter (Packard BioScience).

Na^+^-dependent Pi transport has been shown to be linear up to 60 min upon initiation of uptake, ensuring that uptake per unit time is a measure of transport velocity [39]. Preliminary experiments only with WT hNaPi-IIb and water-injected oocytes as negative controls were performed with an incubation time of 10 and 30 min (Additional file 1: Fig. S3). Based on these data, an incubation time of 10 min was chosen for subsequent experiments including mutants. Three independent experiments were performed for each mutant, always including oocytes expressing WT hNaPi-IIb and non-injected oocytes.

### Western blot and immunocytochemistry

#### Oocyte preparation for western blot

Pools of three oocytes were lysed in 60 μl lysis buffer [100 mM NaCl, 20 mM Tris HCl pH 7.6, 1% Igepal CA 630 (Sigma-Aldrich, Buchs, CH)], by pipetting up and down. To pellet the yolk proteins, the lysates were centrifuged at 16,000*g* for 10 min at 4 °C. After the yolk-free supernatants were collected carefully, avoiding contamination with floating lipids, supernatants were mixed with Laemmli sample buffer (0.38 M Tris base, 8% SDS, 4 mM EDTA, 40% (v/v) glycerol, pH 6.8 with HCl, 4 mg/ml of Bromophenol Blue).

#### Immunoblotting of oocytes homogenates

A volume of the supernatant corresponding to one oocyte was loaded on a 10% acrylamide SDS gel for SDS-PAGE. Separated proteins were transferred from the gel to a polyvinylidene difluoride (PVDF) membrane (Immobilon-P, Millipore, Schaffhausen, Switzerland) in a standard tank system (Mini Trans-Blot, Bio-Rad). PVDF membranes were stained with 0.3% Ponceau in 3% Trichloroacetic acid (TCA) to confirm the transfer and subsequently blocked with Tris-buffered saline (TBS) containing 5% fat-free powder milk for 30 min at room temperature. Membranes were then incubated overnight at 4 °C with primary antibody against c-Myc (M4439, Sigma-Aldrich, Buchs, Switzerland) (1:4000) [40]. After washing with TBS three times followed by blocking, membranes were incubated with anti-mouse secondary antibody linked to horseradish peroxidase (HRP) (GE Healthcare, UK Limited) (1:10,000) for 3 h at room temperature. Membranes were then washed three times with TBS and subsequently exposed to HRP substrate (Western Chemiluminescence HRP Substrate, Millipore, Schaffhausen, Switzerland) for 5 min. Chemiluminescence was detected using a LAS-4000 camera system (Fujifilm).

#### Oocyte preparation for immunocytochemistry

Three days after injection, oocytes were washed with phosphate-buffered saline (PBS). For fixation, eggs were incubated in 3% paraformaldehyde (PFA) in PBS for 6 h at 4 °C. Oocytes were then washed with PBS and incubated in 30% sucrose in PBS overnight at 4 °C for cryoprotection. Hereafter, eggs were incubated in PBS for 15 min at room temperature and then transferred to cryomolds (Cryomold Biopsy, Sakura Finetek Germany GmbH, Staufen, Germany) filled with embedding medium (OCT embedding Matrix, Cell Path, Newtown, United Kingdom). The cryomolds with oocytes were immediately snap-frozen in liquid propane and stored at − 80 °C.

#### Immunostaining

Oocyte cryosections of 5 μm were mounted on slides (Superfrost Plus, Thermo Scientific) and incubated in PBS for 45 min. The slides were then blocked in 1% bovine serum albumin (BSA) in PBS for 15 min at room temperature. After blocking, the slides were incubated overnight at 4 °C with the primary antibody against c-Myc (M4439, Sigma-Aldrich, Buchs, Switzerland) (1:500, 1:1000) diluted in 0.02% sodium azide (NaN_3_) in PBS [41]. The slides were washed twice with hypertonic PBS (18 g NaCl in PBS) and once with PBS, followed by incubation with a donkey anti-mouse Alexa Fluor 594 secondary antibody (1:500, Invitrogen) for 1 h at room temperature. Then sections were washed twice with hypertonic PBS and once with PBS, and coverslips were then mounted with Glycergel (DakoCytomation, Baar, Switzerland). Fluorescence was detected using a Leica fluorescence microscope (Leica CTR600). The freeware programmes Leica AF lite and ImageJ version 1.52a were used to analyse the images.

### Data analysis

The normality of the data was assessed by inspection of QQ-plots. Results are presented as the median and range. Differences between groups were tested with Mann–Whitney *U*-test or Kruskal–Wallis test by ranks. Post-hoc multiple comparisons were performed using Dunn's multiple comparison test. A *p* value < 0.05 was considered statistically significant. Data analysis was performed using Stata 11.2 (StataCorp 2009, College Station, TX, USA).

## Supplementary Information

**Additional file 1** Supplementary Figures S1-S3.** Fig. S1** Phosphate transport activity in *Xenopus laevis* oocytes 3 days after injection of water or cRNA encoding wild-type (WT) c-Myc-hNaPi-IIb. ** Fig. S2** Molecular identification of c-Myc hNaPi-IIb expression by Western Blot (whole blot) of lysates from *Xenopus laevis* oocytes injected with wild-type (WT) and mutated constructs. ** Fig. S3** Final plasmid map of the c-Myc-tagged KSM vector containing hNaPi-IIb cDNA insert.

## Data Availability

The datasets used and analysed in the current study are available from the corresponding author upon reasonable request.

## Abbreviations

BSA: Bovine serum albumin
MS222: Tricaine methanesulphonate
NaPi-IIb: Sodium-dependent phosphate transport protein 2B
NI: Non-injected control oocytes
NMD: Nonsense mediated decay
NS: No secondary antibody
PAM: Pulmonary alveolar microlithiasis
PBS: Phosphate-buffered saline
PFA: Paraformaldehyde
PTC: Premature termination codon
PVDF: Polyvinylidene difluoride
SDS: Sodium dodecyl sulfate
TBS: Tris-buffered saline
TCA: Trichloroacetic acid
TM: Transmembrane
WT: Wild type
