# Supplementary material for: Impaired phosphate transport in SLC34A2 variants in patients with pulmonary alveolar microlithiasis
Source: Hum Genomics. 2022 Apr 20;16:13. doi: 10.1186/s40246-022-00387-y (PMC9019944; doi:10.1186/s40246-022-00387-y)
Supplement: Supplementary file 1 — Additional file 1 Supplementary Figures S1-S3. Fig. S1 Phosphate transport activity in Xenopus laevis oocytes 3 days after injection of water or cRNA encoding wild-type (WT) c-Myc-hNaPi-IIb. Fig. S2 Molecular identification of c-Myc hNaPi-IIb expression by Western Blot (whole blot) of lysates from Xenopus laevis oocytes injected with wild-type (WT) and mutated constructs. Fig. S3 Final plasmid map of the c-Myc-tagged KSM vector containing hNaPi-IIb cDNA insert. [file 40246_2022_387_MOESM1_ESM.pdf]

Supplementary Figures

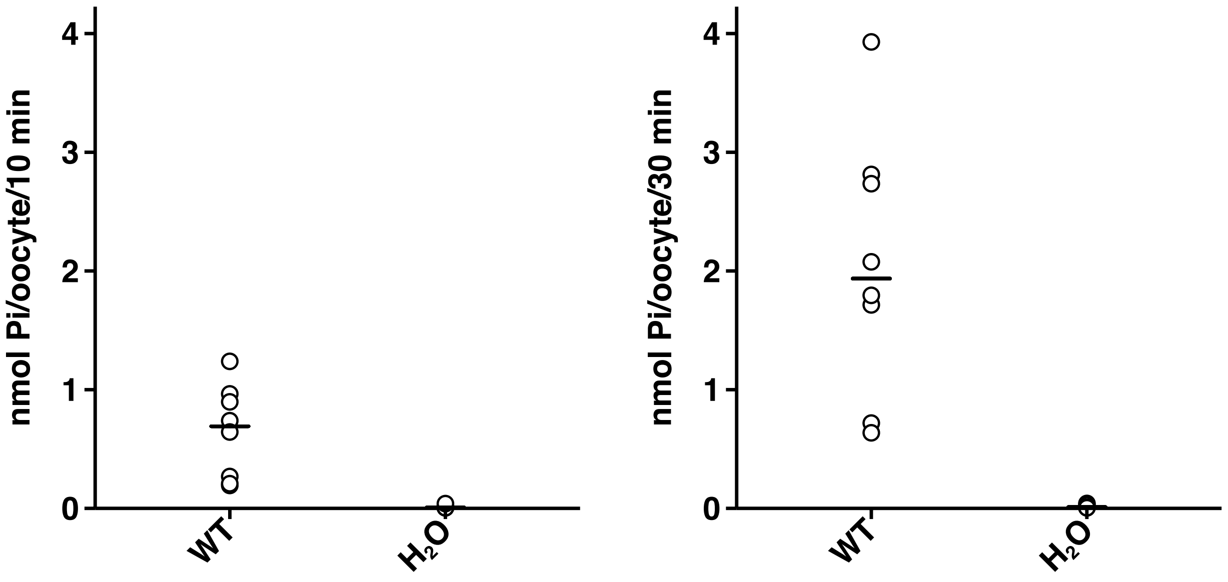

Fig. S1 Phosphate transport activity in *Xenopus laevis* oocytes 3 days after injection of water or cRNA encoding wild-type (WT) c-Myc-hNaPi-IIb. Data are values for measurements from one experiment with 7–8 oocytes per group incubated for 10 or 30 min (left and right, respectively) in ND96 containing 1mM cold Pi and ^{32}P. Vertical bars represent the median transport activity. The WT showed significantly higher ^{32}Pi uptake than water-injected oocytes in both experiments (p = 0.001, Mann-Whitney U-test)

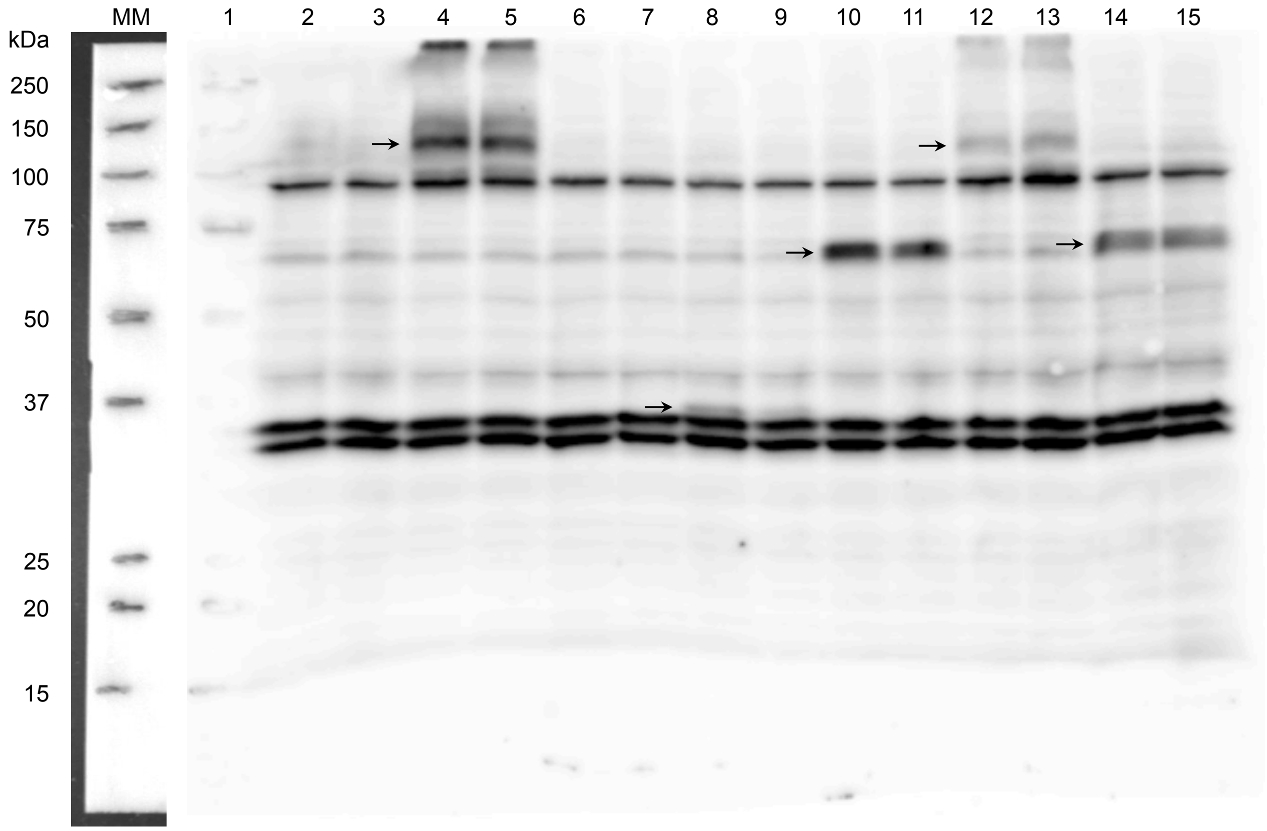

Fig. S2 Molecular identification of c-Myc hNaPi-IIb expression by Western Blot (whole blot) of lysates from *Xenopus laevis* oocytes injected with wild-type (WT) and mutated constructs. Each lane was loaded with a volume corresponding to one oocyte. Lanes correspond to: (1) molecular marker (MM), (2, 3) non-injected control oocytes, (4, 5) wild type, (6, 7) variant not further analysed, (8, 9) Lys304Ter, (10, 11) Leu443fs, (12, 13) Thr468del, and (14, 15) Gln486Ter. Blots were incubated with a monoclonal c-Myc-specific antibody. The approximate protein molecular mass is indicated in kilodaltons (kDa). Specific immunoreactive protein bands are detected at ~110-125 kDa for WT and Thr468del, at ~ 35 kDa for Lys304Ter, and at ~ 60 kDa for Leu443fs and Gln486Ter

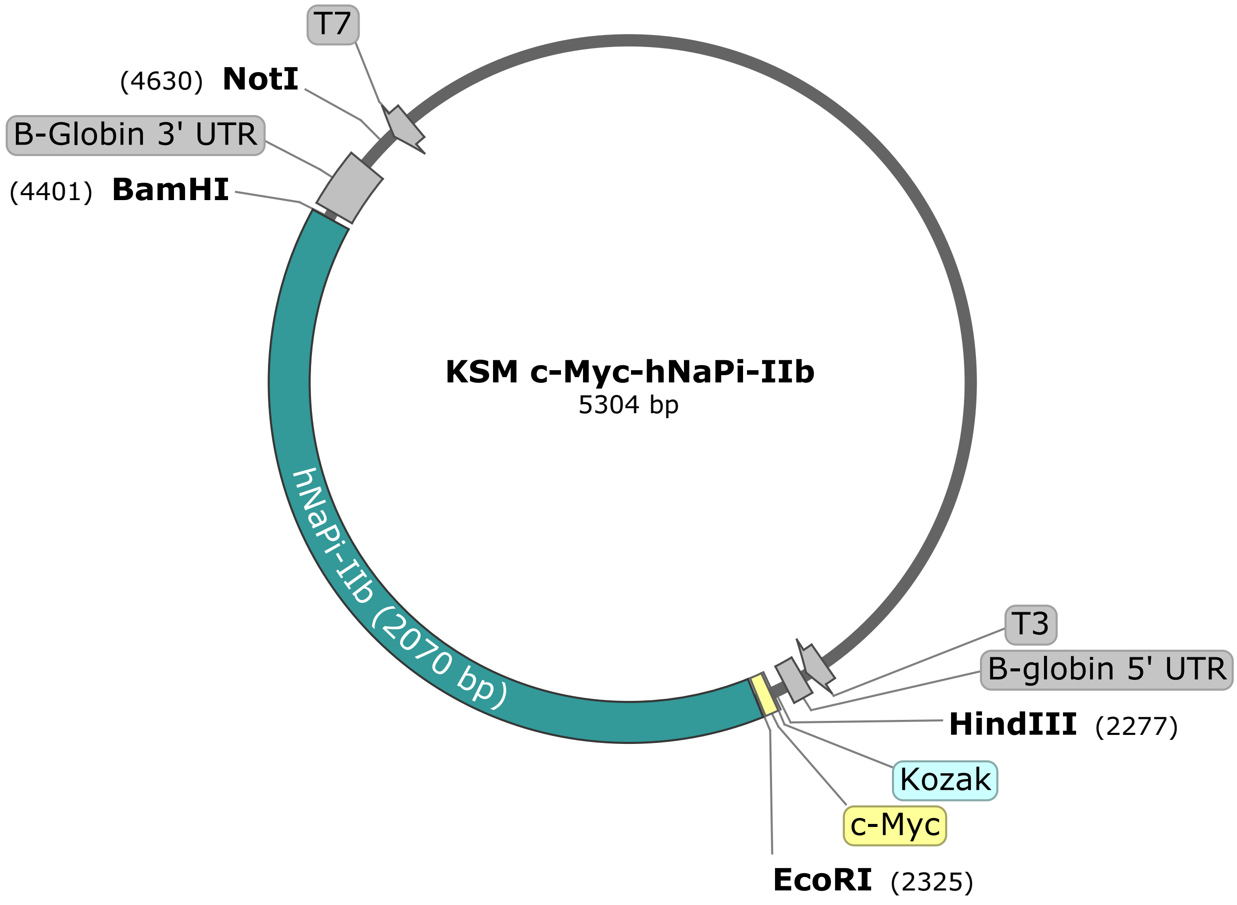

Fig. S3 Final plasmid map of the c-Myc-tagged KSM vector containing hNaPi-IIb cDNA insert. T3 (T3 promoter), T7 (T7 promoter), Kozak (Kozak sequence), c-Myc (c-Myc sequence), hNaPi-IIb (human NaPi-IIb cDNA sequence), HindIII, EcoRI, BamHI, and NotI (recognition sites of HindIII, EcoRI, BamHI, and NotI restriction enzymes)
